# Translation and Validation of the Breast Cancer Awareness Measurement Tool in Malaysia (B-CAM-M)

**DOI:** 10.31557/APJCP.2020.21.1.217

**Published:** 2020

**Authors:** Mila Nu Nu Htay, Michael Donnelly, Desiree Schliemann, Siew Yim Loh, Maznah Dahlui, Nor Saleha Binti Ibrahim Tamin, Saunthari Somasundaram, Tin Tin Su

**Affiliations:** 1 *Department of Social and Preventive Medicine, *; 4 *Department of Rehabilitation Medicine, University of Malaya, *; 7 *National Cancer Society, Kuala Lumpur, *; 2 *Department of Community Medicine, Melaka-Manipal Medical College, Manipal Academy of Higher Education (MAHE), Melaka, *; 8 *South East Asia Community Observatory (SEACO), Monash University Malaysia, Bandar Sunway Malaysia, *; 3 *Centre for Public Health and UKCRC Centre of Excellence for Public Health, Queen’s University Belfast, Belfast, UK, *; 5 *Faculty of Public Health, Universitas Airlangga, Surabaya, Indonesia, *; 6 *Ministry of Health, Putrajaya, Malaysia. *

**Keywords:** Breast cancer awareness- Malay- B-CAM-M- questionnaire validation- cultural adaptation

## Abstract

**Background:**

Breast cancer is the most common cancer among women in Malaysia, and the incidence of 31.1 per 100,000 population is comparatively higher than other Southeast Asian countries. Diagnosis tends to occur at later stages which may be due, partly, to inadequate knowledge about warning signs and symptoms. Therefore, this study investigated the validity and reliability of a UK-developed measure in the context of assessing women’s awareness of breast cancer in Malaysia.

**Aims:**

This study aimed to translate, adapt and validate the internationally recognised Breast Cancer Awareness Measure (B-CAM) into the Malay language.

**Methods:**

The original B-CAM (Cancer Research UK) was forward and backward translated and content validation was ascertained. Face validity (n=30), test-retest reliability (n=50) and the internal consistency of the B-CAM-M (M for Malay language) were assessed in a community sample of adults (n=251) in 2018.

**Results:**

The translated B-CAM-M was validated by an expert panel. The Item-Content Validity Index ranged from .83 to 1.00. The results from the survey (n=251) indicated that the B-CAM-M was well received by Malay-speaking women across the main ethnic groups (85 Malay, 84 Chinese and 82 Indian adults). Cronbach alpha scores for the knowledge about breast cancer symptoms (0.83) and the barriers to healthcare seeking items (0.75) were high. Test-retest reliability (separated by 2-week-interval) with 50 randomly selected participants from the community survey produced intra-class correlations ranging from 0.39 to 0.69.

**Conclusion:**

The Malay-version, the B-CAM-M, is a culturally acceptable, valid and reliable assessment tool with which to measure breast cancer awareness among Malay-speaking women.

## Introduction

Breast cancer is the commonest cancer among women worldwide (WHO, 2014). Similarly, it is the leading cause of cancer among women in Malaysia with an incidence of 31.1 per 100,000 population (Manan et al., 2016). The incidence in Malaysia is higher compared to neighbouring Southeast Asia countries such as Laos, Thailand, Cambodia, Myanmar and Viet Nam (Youlden et al., 2014). Breast cancer is a curable disease if diagnosed at an early stage. However, breast cancer is often diagnosed late, due to lack of awareness, denial, negative beliefs about cancer, reliance on alternative medicine and unawareness about breast cancer screening programs (Abdul Hadi et al., 2010; Al-Dubai et al., 2011; Khan et al., 2015; Yu et al., 2015). There is a need to raise awareness about breast cancer symptoms in Malaysia in order to counter delayed diagnosis. Cancer awareness campaigns intend to transform the attitudes and behaviours of the public and to change their perspectives on traditional and social norms (Schliemann et al., 2018). A number of campaigns and awareness raising events have been organised over the years in Malaysia, however, systematic evaluation of these awareness raising efforts is lacking (Loh et al., 2017). There is a need to create a robust evaluation framework to assess cancer awareness in Malaysia. 

However, a validated assessment tool for use in Malaysia is lacking. A validated tool in Malay language would help to investigate women’s awareness about signs and symptoms and risk factors for breast cancer and the utilization of screening programs as well as contribute to the evaluation of the effectiveness of interventions. The Breast Cancer Awareness Measure (B-CAM) is the internationally recognised measure that was developed (Linsell et al., 2010; CancerResearchUK, 2011) to measure breast cancer awareness in the UK (Campbell et al., 2016). Since then, it has been translated and validated in Arabic, Persian and Swahili (Al-Khasawneh et al., 2016; Heidari and Feizi, 2018; Wachira et al., 2017). 

*Objective*

This study aimed to translate, adapt and validate the internationally recognised B-CAM for use in Malaysia. In addition, findings regarding breast cancer awareness in Malaysia as assessed with B-CAM-M (M for Malay language) affords potential to draw meaningful international comparison with other countries.

## Materials and Methods

The questionnaire validation was based on the original B-CAM developed by Cancer Research UK, King’s College London and University College London (CancerResearchUK, 2011). The permission for the translation and cultural adaptation of B-CAM was granted by the developer (CRUK). Ethical approval was obtained from the University of Malaya Medical Centre (UMMC) Ethics Committee (Ref No. 2016126-4668) and Melaka-Manipal Medical College, Research Ethics Committee. 

*Study design and participants*

The study was conducted in Melaka city, a historical and the fifth largest city in Malaysia. Data was collected between April and May 2018. The sample size of 250 participants was estimated by using the formula of N/p ≥10 (N= sample size, p= number of variable in a model) (Marsh et al., 1998) focusing on the symptoms and barrier domain items. Participants were recruited by using quota sampling with an equal number of women from Malay, Chinese and Indian ethnic backgrounds. Inclusion criteria comprised: women who were aged 40 years and above who could answer independently and who provided written consent. Women who were diagnosed with breast cancer or who were working in health care were excluded. 

*Adaptation of the B-CAM questionnaire*

The B-CAM Toolkit was validated in the UK (Linsell et al., 2010). An expert panel comprising public health specialists (n=2), an expert in instrument development (n=1) and translation and language specialists (n=2) reviewed critically the B-CAM and then adapted and modified the content and response format to be culturally sensitive and appropriate to Malaysians and the local health care system. In addition, the experts (Malay=2, Chinese=1 and Indian=2) amended the content so that it would be acceptable and clear to the ethnic groups in Malaysia. All seven domains of the B-CAM were translated into the Malay language except ‘Knowledge of the NHS Breast Screening Programme’ domain, as this was not applicable to the Malaysian health care system. Breast cancer screening guidelines in Malaysia recommend annual clinical breast examination (CBE) for women aged 40 years or older and biennial mammogram for women aged between 50 and 74 years (CPG, 2010). Therefore, the following two items were added to the modified version in line with the Malaysia breast cancer screening program ‘Were your breasts examined by trained health professionals such as doctors or nurses within one year?’ and ‘Have you had a breast cancer screening (mammogram) in the last two years?’. In the risk factor domain, the following risk factors were explained: one alcohol unit, having children early on in life, early menarche, late menopause and moderate physical activity. The other domains were translated into Malay without changing the original items. 

*Linguistic validity*

The English version of the B-CAM was translated to Malay by using the forward and backward translation method following the World Health Organisation (WHO) guideline (WHO, 2015). The professional translator translated the conceptual equivalent of the questions by using culturally acceptable Malay words and phrases. Three rounds of harmonization checked the content, concepts and discrepancies between the original English version and the forward-translated Malay version. Firstly, the translated Malay version was reviewed by the five-person independent bilingual expert panel. Secondly, a group discussion was conducted with eight bilingual experts. Lastly, harmonisation was completed via appraisals that were conducted by a bilingual expert and an occupational therapist educator. The final Malay version was backward translated to English by a bilingual academician. The backward translated English version of the questions and the original English versions were checked for conceptual and cultural equivalence. 

*Content validity*

Content validity was examined in consultation with the expert panel. The content validation experts were independent and different from those experts who participated in the linguistic validation. The English and Malay versions of the questionnaire were assessed by six bilingual experts who were oncologist (n= 1), a psychiatric doctor (n=1) and primary health care physicians (n= 4) and the Item-Content Validity Index (I-CVI) was calculated based on their ratings. The items which achieved I-CVI of ≥ .85 were included in the questionnaire. 

*Face validity*

Face validation was carried out with 30 participants, 10 in each ethnic group (Malay, Chinese and Indian). At the end of the interview the participants were asked questions regarding the quality, readability and understanding the questionnaire. 

*Test-retest reliability*

Reproducibility of the B-CAM-M was assessed through the test-retest method. The sample size was calculated with alpha of 0.05 and power 90%, to detect the ICC value of .30 targeting for three awareness scores, while considering 25% of attrition rate (Bujang and Baharum, 2017) The test-retest reliability was conducted with 50 randomly selected participants from the community survey. For the second visit, the enumerators called the selected participants and arranged appointments. These participants were interviewed for the second time by using the same questionnaire with an interval of 2 weeks after the first visit. 

*Scoring and statistical analysis*

The B-CAM-M included 11 items about warning signs and symptoms of breast cancer. Nine out of 11 items asked about symptoms other than lumps. Participants who responded “Yes” to five or more questions about non-lump symptoms were considered to be “aware of the warning signs” of breast cancer (Linsell et al., 2010); and participants who responded that, “50 year-old women are more likely to get breast cancer in the next year” were considered to be aware of age-related risk. Reported breast self-examination once a week or once a month was considered to denote awareness of the value of self-examination for any changes in the breast. Therefore, the total awareness score ranged from 0 to 3 (Linsell et al., 2010). 

The data were analysed using PASW (version 18) statistical software. Descriptive analysis was carried out for the participants’ demographic variables. The content validation was assessed by computing the Item- Content Validity Index (I-CVI). Reliability analysis was conducted by the test-retest method by computing the intraclass correlation (ICC) and internal consistency by Cronbach’s alpha. 

## Results

*Socio-demographic characteristics*

A total of 281 participants (30 for face validation and 251 for community survey) were recruited for this study. The ethnic distribution of participants in the community survey (n=251) was 33.9% Malay, 33.5% Chinese and 32.6% Indian (Table 1). Approximately 50% of the participants obtained secondary education. The majority of the participants (82.9%) were married women. Among the participants, 39% of women were homemakers/ housewives (unemployed) and 43.8 % were either service and sales workers, professionals or clerical support workers. Approximately half of the participants (54.6%) had a monthly family income of less than RM 3,000, which is considered as the bottom 40% income group in Malaysia (DOSM, 2017) (Table 1). 

*Adaptation and Linguistic validation*

Regarding knowledge about signs and symptoms items, the experts advised use of the word “ketulan” rather than “benjolan” for the most accurate translation of ‘breast lump’. The English phrase of “puckering or dimpling of the breast skin”was amended to read as “kedutan atau kulit mengelupas di kulit payudara” in order to be appropriate and accurate in Malay language. In the risk factors domain, “Having children later on in life or not at all” was amended to read as “Melahirkan anak pada usia yang lanjut atau tiada pernah melahirkan anak”. 

*Content validation*

According to the protocol, we invited nine bilingual experts who were public health specialist, oncologists and clinicians. However, only six experts responded and provided their opinions about the items for content validation. The I-CVI was calculated based on the rating for each item that was provided by the experts. The I-CVI of all 50 items in this questionnaire ranged between 0.83 - 1.00. 

*Face validation*

Thirty participants, ten from each ethnic group, were interview for the face validation. The participants responded that all the questions were understandable, acceptable in Malaysian culture and they had no objections to answer all the questions. Some participants noted that “Worried about wasting the doctor’s time” might not be a barrier in this context as the community believed that devoting time for a patient was a doctor’s responsibility. 

*Distribution of responses to the B-CAM-M*

A total of 251 participants were recruited for the face-to-face interview and asked to complete the B-CAM-M. The majority of participant were aware of lump as a symptom (90%), discharge (80.5%) and pain (75.3%). ‘Worrying about what the doctor might find may stop me from going to the doctor’ was the commonest barrier reported (37.4%). Approximately half of participants admitted that they performed regular breast self-examination, i.e. once a week or once a month (52.2 %) (Table 2).

*Test-retest reliability*

Open-ended questions were excluded from the test-retest reliability analysis. In the individual item analysis, 19 items had an ICC value of < 0.4, 15 items had an ICC value between 0.41 - 0.69 and four items had an ICC value >0.7. The composite score for breast cancer awareness was calculated for the participants and the ICC value for test-retest reliability analysis was 0.79 (Table 3). 

*Internal consistency*

The internal consistency was assessed by calculating Cronbach’s alpha. The alpha coefficient for the items regarding knowledge about breast cancer symptoms was 0.83 and 0.75 for items regarding barriers to healthcare seeking (Table 4). 

**Table 1:** Demographic Patterns of Participants (n=251)

Variables	n	%
1. Ethnicity		
Malay	85	33.9
Chinese	84	33.5
Indian	82	32.6
2. Religion		
Islam	86	34.3
Hinduism	75	29.9
Buddhism	74	29.5
Others	16	6.3
3. Marital Status		
Married	208	82.9
Single	42	16.7
No reply	1	0.4
4. Number of household members		
1- 5 members	169	67.3
6- 10 members	78	31.1
More than 10 members	1	0.4
No reply	3	1.2
5. Educational level		
No formal education	7	2.8
Primary education	56	22.3
Secondary education	123	49.0
Tertiary education	61	24.3
Others	4	1.6
6. Current Job Status		
Employed	137	54.6
Unemployed (homemakers/ housewives)	113	45
No reply	1	0.4
8. Monthly family income		
Below RM 3, 000	137	54.6
RM 3, 000 – RM 5, 000	58	23.1
Above RM 5, 000	46	18.3
No reply	10	4.0

**Table 2:** Distribution of Participants Responses to the BCAM (n=251)

Domains	Responses on “Yes” or “Agree”	n	%
Knowledge on breast cancer signs and symptoms	Lump or thickening in breast	226	90.0
	Discharge or bleeding from nipple	202	80.5
	Lump or thickening under armpit	199	79.3
	Pain in breasts or armpit	189	75.3
	Changes in shape of breast or nipple	180	71.7
	Changes in size of breast or nipple	171	68.1
	Redness of breast skin	156	62.2
	Pulling in of nipple	139	55.4
	Change in the position of nipple	131	52.2
	Nipple rash	131	52.2
	Puckering or dimpling of breast skin	128	51.0
Barriers for breast cancer screening	Worrying about what the doctor might find may stop me from going to the doctor	94	37.4
	Too many other things to worry about	64	25.5
	Too scared	59	23.5
	Too embarrassed	54	21.6
	Too busy to make time to go to the doctor	51	20.4
	Difficult to make an appointment with doctor	35	14.0
	Not feeling confident talking about my symptom with the doctor	29	11.6
	Difficult to arrange transport to the clinic	28	11.2
	I find my doctor difficult to talk to	22	8.8
	Worried about wasting the doctor’s time	7	2.8
Knowledge on risk factors	Past history of breast cancer	159	63.3
	Using HRT (Hormone Replacement Therapy)	142	56.6
	Having a close relative with breast cancer	140	55.8
	Doing less than 30 minutes of moderate physical activity 5 times a week	127	50.6
	Drinking more than 1 unit of alcohol a day	95	37.8
	Being overweight	94	37.5
	Having children later on in life or not at all	85	33.9
	Having a late menopause	57	22.7
	Starting your periods at an early age	45	17.9
Age-related risk	A 30 year old woman	75	29.9
	A 50 year old woman	71	28.3
	A 70 year old woman	4	1.6
	A woman of any age	88	35.1
	No reply and Don’t know	13	5.2
Breast self-examination	Rarely or never	80	31.9
	At least once every 6 months	40	15.9
	At least once a month	55	21.9
	At least once a week	76	30.3
Attendance on breast cancer screening	Breast examination by trained health professional within one year	112	44.6
	Had mammogram in the last 2 years	86	34.3

**Table 3:** Intra-Class Correlation of Knowledge on Breast Cancer Symptoms, Age-Related Risk and Breast Self-Examination

No.	Domain	Item numbers	ICC	95% CI	F test	Significant level
1	Knowledge of symptoms (non-lump symptoms)	No 11-15, 17-18, 20-21	0.39	-0.071-0.655	1.646	0.42
2	Knowledge on age related risk	No 37	0.69	0.449-0.823	3.2	0
3	Frequency of BSE	No 22	0.53	0.178-0.735	2.144	0
	Total scoring for breast cancer awareness (score 0-3)		0.79	0.630-0.881	4.757	0

**Table 4:** Internal Consistency (Cronbach’s Alpha) of Subscales in the B-CAM-M

No.	Domain	Total number of items	Cronbach’s alpha
1	Knowledge of symptoms	11	0.83
2	Barriers for health care seeking	10	0.75
	Both symptoms and barriers domains	21	0.73

## Discussion

This study developed a tool to measure awareness on breast cancer adopting a tool used in UK, and adapting to suit the local culture and context. The validation process showed that the majority of items in the original B-CAM were applicable to Malaysians suitably translated and adapted and only a small number of items, specifically referring to the local health care system were changed. 

A rigorous linguistic validation amended the initial forward-translated measure to become a more accurate and culturally acceptable instrument. Since Malaysia is interwoven with a multicultural and multilingual community, input from Malay Chinese and Malay Indian during the process of linguistic validation were ascertained in order to prepare an acceptable and understandable questionnaire tool for different ethnic groups. Bahasa Malaysia (BM) is the official language in Malaysia, and therefore the translation process was focused on the BM. Meanwhile, the Malay terms were carefully checked and revised by the experts to maintain the accuracy of the meaning that is conveyed in the original B-CAM and to avoid the cultural sensitivity.

The translated B-CAM-M was reviewed by an expert panel of oncologist, psychiatric doctor and primary health care physicians, and they provided ratings regarding, for example, the relevancy of the items and advice about how to establish content validity. According to Lynn (1986), an item scored .85 was considered to be acceptable for inclusion in a questionnaire (Lynn, 1986). All 50 items in the modified and translated B-CAM indicated that they met this criterion and that they were relevant to be included in the tool. 

During the face validation process, the women’s responses and very low rate of missing data proved that the B-CAM-M questions were clear and that there was no objection, culturally or otherwise, to answering the questions. The team decided to retain the item about ‘wasting a doctor’s time’ in order to test its value in the community survey. The analysis of the community survey data revealed that the item had the lowest score among all the barrier items. 

The internal reliability of the B-CAM-M was measured by Cronbach’s alpha, and the findings suggested that there was high internal consistency between the items regarding knowledge about breast cancer symptoms (.83). This finding is in keeping with previous translation and validation studies of B-CAM in Arabic (Cronbach’s alpha: .89), and Persian languages (Cronbach’s alpha: .88) (Al-Khasawneh et al., 2016; Heidari and Feizi, 2018). 

Test-retest reliability was checked by conducting a second-time interview with 50 participants after two weeks. According to Fleiss (1986), the ICC value of .40 to .75 is considered to be “fair to good” (Fleiss, 1986). Some items from the domains of symptoms, risk factors and barriers had ICC values lower than 0.4 and discrepancies mainly occurred with respect to participants’ knowledge and barriers for health care seeking. In comparison, a higher ICC value of 0.84 was reported for warning signs in the Persian version (Heidari and Feizi, 2018). A possible explanation for the lower ICC value might be related to the fact that although participants were not informed that they would be asked the same questions, some participants may have searched for information about breast cancer from the media and websites due to their interest and curiosity being stimulated by the first interview. During the validation process of the original B-CAM, Linsell et al reported lower ICC values ranging from 0.28 – 0.68 as a reason for the improvement in scoring the second time of applying the questionnaire (Linsell et al., 2010). None the less, the overall knowledge score, age-related risk score and breast self-examination score of B-CAM-M had moderate to good ICC (0.39 to 0.69). Therefore, all the items were maintained in the questionnaire after the analysis. 

Among the Malay population, the Bahasa Malaysia can also be different because of dialects in different states. Our validation study was conducted with Malay, Indian and Chinese ethnic groups in Malaysia, and therefore indicates that the questionnaire measure appears to be acceptable across the different Malay-speaking ethnic groups. The instrument was considered to be easy to understand though more than 70% of participants completed secondary education and, so, it is unclear how well the questionnaire would work with people who have not completed secondary education or who have low levels of literacy. However, the questionnaire measure appeared to work well with people across all income brackets including low-income earners.

A limitation of this study was that the participants were recruited by quota sampling method. However, quota sampling provided to recruit approximately equal number of participants from the three major ethnic groups (Malay, Chinese and Indian) in Malaysia. 

In conclusion, the B-CAM-M is culturally acceptable and valid among the Malay-speaking different ethnic groups in Malaysia. This tool may be used to provide a valid assessment of breast cancer awareness in the Malaysian context. Understanding breast cancer awareness is important for guiding public health authorities and policy makers to implement effective strategies to improve awareness of symptoms and utilisation of breast cancer screening services. In addition, this tool will be useful for the evaluation of future awareness raising interventions and other public health programs. 

*Implications for Practice*

The culturally acceptable, valid and reliable B-CAM-M tool could help to assess the breast cancer awareness level in the community and to evaluate the effectiveness of awareness raising interventions. 

## Abbreviations

B-CAM, Breast Cancer Awareness Measure; B-CAM-M, Breast Cancer Awareness Measure-Malay Language; CRUK, Cancer Research UK; WHO, World Health Organization; MOH, Ministry of Health, Malaysia; CBE, Clinical Breast Examination; I-CVI, Item-Content Validity Index; ICC, Intraclass correlation.
